# Review of *Genitori cercasi. L’Italia nella trappola demografica* edited by Letizia Mencarini and Daniele Vignoli

**DOI:** 10.1186/s41118-018-0049-3

**Published:** 2018-12-21

**Authors:** Massimo Livi-Bacci

**Affiliations:** University of Florence, Florence, Italy

## Abstract

Letizia Mencarini e Daniele Vignoli, Genitori cercasi. L’Italia nella trappola demografica [Parents Wanted. Italy in the Demographic Trap], Milano: Egea; 2018. 149 pages, ISBN 978-88-8350-279-8

This well-written, clearly articulated, and documented book of Mencarini and Vignoli deals with one of the central questions of many contemporary populations: the “birth dearth,” fruit of a downward spiral of low fertility, declining number of potential parents, and declining number of births. The mechanisms of this negative spiral, or demographic trap, in the authors’ definition—1.035 million babies were born in Italy in 1964, more than double of the 0.458 million of 2017—are well known to the specialists, but remain obscure to the general public. This widespread ignorance is also the consequence of the failure of specialists to disseminate and communicate to the general public the results of their research, leaving this vital function in the hands of the media.

In Chapter 1, the Italian demography is compared with that of the major European countries. Italy, with Spain, has the lowest fertility, the highest mean age of mothers at the birth of their children, the highest life expectancy, and the larger excess of deaths over births. Germany, that shared a demographic situation similar to that of Italy and Spain, has undergone an impressive recovery of fertility in the last few years. The demography of France and of the UK is, on the other hand, in a situation of equilibrium. Chapter 2 retraces the course of fertility after the postwar recovery until the 1960s, and the continuous decline from the 1970s to the turn of the century, the illusionary mini-recovery in the first decade of the new millennium, and the further decline during the Great Crisis. Much of the decline has been the consequence of a continuous postponement of the first birth and of the substantive increase of the mean age at childbearing. Fertility decline is smoother when cohorts are observed, with the number of children per woman falling to 1.6 for cohorts born around 1970. Childlessness, for the same cohorts, has increased to over 20%, often because of a conscious decision of mothers or of a prolonged postponement of the decision to have a first baby. In the last decade—in Italy, the crisis of the economy has been more severe than elsewhere—the age structure of women in reproductive age has changed as the numerous women born during the 1960s and 1970s have been replaced by the increasingly less numerous ones born in the 1980s and 1990s, when total fertility had fallen consistently below 1.5 (Chapter 3). The authors conclude that the postponement of births in the life cycle and the decline of the numbers of potential mothers have had a depressive effect on births larger than that determined by the crisis and by the worsening of the standard of living. So the “trap” has been closing up, low fertility has produced a declining number of potential mothers, and this downward trend is set to continue in the future, with a negative effect on the number of births, even if a recovery takes place as hypothesized by current population projections (Chapter 4). A recovery of fertility is in the realm of possibilities, also because surveys consistently show that Italian women, and men, desire or consider ideal a number of children around two, the largest gap between aspirations and realization in European countries (Chapter 5). But why Italian fertility is so low? (and in South Korea, Japan, Spain, Poland, and many other populations, one should add). According to the authors, the traditional explanations based on the cost-benefit analysis of Beckerian inspiration, or on the “second demographic transition” paradigm, seem to lose strength in the “postmodern” societies of the new millennium (Chapter 6). Although delayed, the many transformations that have changed the traditional identity of the family in Europe since the 1970s are taking hold in Italy: more women at work, more unions and less marriages, an increasing number of children born outside the marriage, and more divorces and separations. But this transition has failed to be sustained by an adequate transformation and adaptation of the welfare system, and, above all, by an egalitarian revolution in gender roles, and more so inside the family than in society at large.

Mencarini and Vignoli are not sanguine about the future, although they agree that a mix including a reform of the welfare system, efficient policies sustaining women in the labor market (also in Italy, there is some evidence that fertility is higher where more women are at work), immigration, and (perhaps above all) more egalitarian gender relations might help the country to open the “demographic trap” in which is the prisoner. One factor equally important in determining the structural low fertility, and that the authors do not mention, is the long permanence of children in the family and the delay in which the full economic autonomy is reached by young people, and, as a consequence, the delay in taking crucial decisions—such as forming a stable union or having a child. After all, in a cost-benefit approach, the cost of a child that becomes autonomous at 20 is much lower than the cost of a child that leaves the parents’ house at 30!

Policymakers’ attention rapidly decays the farther the horizon of a decision is. But, as Mencarini and Vignoli rightly say, before forming a decision and taking action, it is necessary to know the facts. Now, thanks to this book, the facts are clear and the cards are on the table. But will policymakers play the game?

Massimo Livi Bacci

26 XI 2018

